# Cervical Precancerous Lesions and Associated Factors Among Women Screened in Two Hospitals in the City of Douala, Cameroon

**DOI:** 10.7759/cureus.41993

**Published:** 2023-07-17

**Authors:** Richard Tagne Simo, Claude Verdiane Mbock, Armel Herve Nwabo Kamdje, Arsène Godlove Djoko Nono, Charlette Nangue, Phelix Bruno Telefo

**Affiliations:** 1 Department of Biomedical Sciences, University of Ngaoundere, Ngaoundere, CMR; 2 Department of Biochemistry and Physiology, University of Garoua, Garoua, CMR; 3 Department of Biomedical Sciences, Catholic University of Cameroon, Bamenda, CMR; 4 Department of Biochemistry, University of Dschang, Dschang, CMR

**Keywords:** precancerous cervical lesions, hsil, hpv, risk factors, screening

## Abstract

Introduction: Cervical cancer remains the second leading cause of death among women in Cameroon despite the new strategies put in place. This study was conducted in order to determine the prevalence of precancerous cervical lesions and its associated factors in Douala (Cameroon).

Methods: A cross-sectional study was conducted over a period of nine months in two hospitals of the city of Douala, Cameroon (Laquintinie Hospital and Gyneco-Ostetric and Pediatric Hospital). Cervico-vaginal and endocervical samples were taken from women attending the above-mentioned hospitals in order to identify and characterize precancerous lesions by cytological examination and to genotype for human papillomavirus (HPV) using the Abbott RealTime High-Risk (HR) HPV kit. Data of sociodemographic characteristics, clinical history, and knowledge about cervical cancer were collected using a questionnaire.

Results: Of the 196 women included in this study, 17% had precancerous lesions, including 1.53% for atypical glandular cells (AGC), 4.53% for atypical squamous cells (ASC), 4.53% for low-grade squamous intraepithelial lesion (LSIL), 5.61% for high-grade squamous intraepithelial lesion (HSIL), 0.51% for atypical squamous cells of undetermined significance (ASC-US), and 0.51% for atypical squamous cells cannot exclude HSIL (ASC-H). In addition, the prevalence of HPV infection was 18%, of which 2% was for HPV 16, 2% for HPV 18, and 14% for undetermined HPV. A positive association was recorded between the occurrence of precancerous lesions and HPV infection (*P=0.01*), age, and school level. Moreover, the occurrence of precancerous lesions was positively associated with the participants' level of knowledge (P=0.01).

Discussion: Precancerous lesions were predominantly HSIL, and the factor most associated with these lesions was HPV infection.

Conclusion: This study demonstrates that diagnosis is made at a relatively late stage due to a low level of knowledge about cervical cancer in the population.

## Introduction

Cervical cancer is the result of the excessive and uncontrolled proliferation of the cervical cells of the cervix. It is secondary to a persistent infection by the human papillomavirus (HPV) [1], which is transmitted through sexual intercourse and can later cause cervical cancer through a slow growth over a period of 10-20 years [2]. The global incidence of cervical cancer is greater than 530,000 annually, with death approaching 275,000 per year [3]. The prevalence of cervical cancer worldwide is estimated by Saslow et al. [4] to be 12%, and almost 90% of deaths due to cervical cancer occurred in developing countries [5]. The majority of the disease burden (about 85%) occur in Sub-Saharan Africa (SSA) [6,7], accounting for about 20.8% of all cancers in women and 14.2% of all cancer-related deaths in women. In Cameroon, the disease is the second most common cancer in women and the leading cause of cancer-related deaths [8]. Cervical cancer generally begins with precancerous lesions or cervical dysplasia, which makes it one of the cancers that can be prevented, cured, or even eradicated, notably through vaccination, screening, and treatment of precancerous lesions [9]. Compliance with certain guidelines, including early screening, has made it possible to reduce the incidence of cervical cancer by 90% in developed countries, in contrast to developing countries where the incidence is constantly increasing. This occurs despite the establishment of cervical cancer control strategies and programs. The mortality rate is worrying because 62.9% of diagnoses are made at a very advanced stage [10], and anatomopathological examinations are found only in the cities of Yaoundé and Douala. As a result, people diagnosed late usually die within 12 months [11].

The aim of this research was to determine the prevalence of precancerous cervical lesions and associated factors at the Laquintinie Hospital and Gyneco-Obstetric and Pediatric Hospital in the city of Douala, Cameroon. A large number of cases from all social groups and classes use the facilities of the hospitals mentioned above, making them suitable for the study.

## Materials and methods

Participants and study procedure

The study was carried out at the Laquintinie Hospital and Douala Gyneco-Obstetric and Pediatric Hospital in Douala over a period of nine months. Our sample size was 329 women to be recruited using the non-probability exhaustive sampling method. Thus, any women presenting to the clinical biology and anatomopathology laboratories of these facilities was included in our study population. However, in terms of selection criteria, women aged 18 and over who were sexually active and consented to take part in the study were included. Women who had undergone partial or total hysterectomy were excluded.

Cervical Specimen Collection and Cytology

Cervical samples were collected using standard gynecological procedures. Briefly, two exfoliated cervical cell specimens were collected for HPV DNA genotyping assays and liquid-based cytological diagnosis, with a cytobrush and an Ayres spatula. Sample slides were prepared using a liquid-based cytology (BD SurePath™ liquid-based Pap test, Becton, Dickinson and Company (BD), New Jersey, USA) method, and slides were read and interpreted according to the Bethesda 2001 classification: (i) normal cases may include reactive change due to inflammation, fungal infection, and atrophy; and (ii) cytological cases of squamous cell carcinoma (SCC), high-grade squamous intraepithelial lesion (H-SIL), atypical squamous cells (ASC) cannot exclude H-SIL (ASC-H), a low-grade squamous intraepithelial lesion (L-SIL), and typical squamous cells of undetermined significance (ASC-US).

DNA Extraction and Genotyping

Genotyping was based on the detection of the viral genome and the determination of the different HR-HPV types. This test was performed in the virology laboratory of the International Reference Centre Chantal Biya (CIRCB), and the method used was real-time polymerase chain reaction (PCR) using the Abbott RealTime HR HPV kit (Abbott Molecular, USA). At the time of testing, 600 µL of each sample was aliquoted and transferred to haemolysis tubes. The Abbott RealTime HR HPV kit uses the m2000sp instrument (Abbott Molecular, USA) for DNA extraction and the Abbott m2000rt instrument for HPV amplification and detection.

A primer mix consisting of 3 sense and 2 anti-sense primers targeting a conserved L1 region was used to amplify HPV targets. The signal for 14 HR-HPV genotypes (HPV 16, 18, 31, 33, 35, 39, 45, 51, 52, 56, 58, 59, 66, and 68) was generated using fluorescently labelled probes. Internal control amplicons (in the aim of validate sample extraction and amplification efficiency) were generated using a primer pair targeting an endogenous human β-globin sequence and were detected using the internal control specific probe. Probes for HPV 16, HPV 18, non-HPV 16/18 (other HR-HPV), and internal control genotypes were labelled with a different fluorochrome, allowing their signals to be recognized in a single reaction.

Determination of Factors Associated With Precancerous Lesions

A questionnaire was made available to the women included in the study to collect information on sociodemographic data, gynecological history, and knowledge of cervical cancer. The answers provided allowed us to determine the etiology of precancerous lesions.

Data Analysis

The survey forms were tabulated, analyzed, and processed. A chi-square test was used to compare frequencies, and P<0.05 was considered statistically significant. All statistical calculations were performed using SphinxPlus.V5.Tuite (Le Sphinx, France) and Microsoft Excel 2013 (Microsoft Corporation, USA).

Ethical approval

Qualified women for the study were informed on the reason, detailed information, and procedures associated to the study. Only women willing to join the study and to sign the information consent form were included. All procedures were approved by the Institutional Ethics Committee of the University of Douala, Cameroon, through authorization N° 2501 CEI-Udo/07/2021/M and the Ethics Committee of Gyneco-Obstetric and Pediatric Hospital in the city of Douala through authorization N° 2021/0065/HGOPED/DG/CEI. All information was treated with strict confidentiality.

## Results

Sociodemographic characteristics

Of the 340 women pre-selected for the study, 196 signed the inform consent. Seven were excluded due to unsuitable sample slides, and 189 women were included. As presented in Table 1, the most represented age groups were 40 to 49 years, accounting for 34.70%. The majority of the women (66.84%) were married, working in the informal sector (53.57%), and had a secondary education (51.02%).

**Table 1:** Sociodemographic characteristics of the women screened for the study

Variables	N (%)
Marital status	
Single	65 (33.16)
Married	131 (66.84)
Age	
20-29	24 (12.24)
30-39	65 (33.16)
40-49	68 (34.69)
50-59	27 (13.78)
60-69	8 (4.08)
≥70	4 (2.04)
Profession	
Formal	86 (43.88)
Informal	101 (51.53)
Unemployed	9 (4.59)
Educational level	
Did not attend school	5 (2.55)
Primary	18 (9.18)
Secondary	100 (51.02)
University	73 (37.24)

Characterization of precancerous lesions and HPV genotyping

The distribution of precancerous lesions (Table 2) in this study population shows that 17.25% of the women had precancerous lesions, in which 5.61% were high-grade lesions and 11.73% were low-grade lesions (1.53% atypical glandular cells (AGC), 4.59% ASC, 0.51% ASC-US, 0.51% ASC-H, and 4.59% LSIL).

**Table 2:** Types of precancerous lesions classified according to the 2001 Bethesda System NIL/M: no intraepithelial lesion or malignancy; AGC: atypical glandular cells; ASC: atypical squamous cells; ASC-US: atypical squamous cells of undetermined significance; ASC-H: atypical squamous cells cannot exclude HSIL; HSIL: high-grade squamous intraepithelial lesion; LSIL: low-grade squamous intraepithelial lesion

Cytological observation	N (%)
NIL/M	156 (79.59)
AGC	3 (1.53)
ASC	9 (4.59)
ASC-US	1 (0.51)
ASC-H	1 (0.51)
HSIL	11 (5.61)
LSIL	9 (4.59)

**Figure 1:**
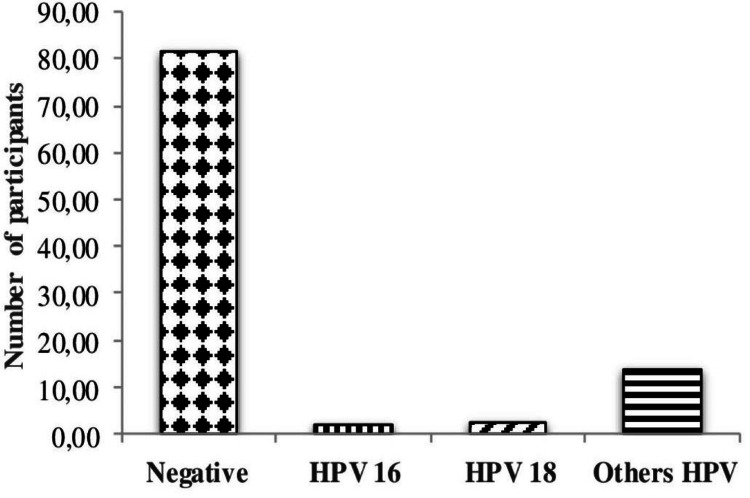
HPV genotypes present in the study population HPV: human papillomavirus

The study on the HPV type distribution presented predominance of negative women (82%). Among the 18% women infected with HPV, 2% were infected with genotype 16, while 16.2% were infected with genotype 18, and the remaining 14% were infected with genotypes other than 16 and 18 (Figure 1).

Investigation of the distribution of precancerous lesions according to HPV genotypes (Table 3) shows that among the 34 women who developed precancerous lesions, 27 were negative and the remaining seven were predominantly infected with HPV genotypes other than 16 and 18. Indeed, of the 156 women without lesions, 128 were negative and 28 were HPV positive, of which 21 were infected with HPV other than 16 and 18.

**Table 3:** Classification of HPV genotypes by cytopathology HPV: human papillomavirus; NIL/M: no intraepithelial lesion or malignancy; AGC: atypical glandular cells; ASC: atypical squamous cells; ASC-US: atypical squamous cells of undetermined significance; ASC-H: atypical squamous cells cannot exclude HSIL; HSIL: high-grade squamous intraepithelial lesion; LSIL: low-grade squamous intraepithelial lesion

Lesions	HPV genotypes			
	Negative	HPV 16	HPV 18	Others HPV
NIL/M	128	3	4	21
AGC	2	0	1	0
ASC	7	0	0	2
ASC-US	0	0	0	1
ASC-H	0	1	0	0
HSIL	10	0	0	1
LSIL	8	0	0	1
TOTAL	155	4	5	26

History of screening

Table 4 presents the patient history of the Pap test. It shows that 61.73% of the women have never been screened for cervical cancer, the most mentioned reasons being lack of information (34.23%), negligence (32.43%), and not being prescribed by a medical staff (20.72%). As for those who did (35.71%), most of them did it only once (61.43%) mainly for medical follow-up (44.29%).

**Table 4:** Distribution of participants according to whether they had been screened before the study period

Variables	N(%)
Cervical screening	
Yes	70 (35.71)
No	121 (61.73)
Reasons	
Medical follow-up	31 (44.29)
Voluntary screening	24 (34.29)
Campaign	7 (10)
Medical advise	8 (11.43)
Carrying out cervico-vaginal smears (CVS)	
One	43 (61.43)
Two	14 (20)
>Two	13 (18.57)
Absence of screening	
Lack of information	38 (34.23)
Negligence	36 (32.43)
Not prescribed	23 (20.72)
Fear	7 (6.31)
Absence of time	3 (2.70)
No financial means	1 (0.90)
Excluded	1 (0.90)
No opportunity	1 (0.90)
Infrequent sexual intercourse	1 (0.90)

The study on the impact of sociodemographic characteristics on the participants' knowledge about cervical cancer and the development of precancerous lesions in women (Table 5) presents a positive correlation between three factors (age, occupation, and education) and precancerous lesions (P=0.01). Furthermore, it shows a highly significant positive association between the participants' occupation, their level of education, and their level of knowledge (P=0.01).

**Table 5:** Impact of sociodemographic factors on precancerous lesions and the level of knowledge L1: no answers; L2: no intraepithelial lesion or malignancy (NIL/M); L3: atypical glandular cells (AGC); L4: atypical squamous cells (ASC); L5: atypical squamous cells of undetermined significance (ASC-US); L6: atypical squamous cells cannot exclude HSIL (ASC-H); L7: high-grade squamous intraepithelial lesion (HSIL); L8: low-grade squamous intraepithelial lesion (LSIL); K1: no knowledge, K2: low knowledge; K3: medium knowledge; K4: high knowledge

Variables	Precancerous lesions										Knowledge about cancer					
	L1	L2	L3	L4	L5	L6	L7	L8	p-value	χ ^2^	K1	K2	K3	K4	P-Value	χ^2^
Age																
20-29	0	23	0	1	0	0	0	0	0.01	0.94	8	6	5	5	0.3	16.90
30-39	4	52	0	3	1	0	1	5			24	18	15	8		
40-49	2	56	3	3	0	0	2	2			25	29	10	4		
50-59	1	19	0	1	0	0	4	2			14	9	3	1		
60-69	0	5	0	1	0	0	2	0			5	1	1	1		
≥70	0	1	0	0	0	1	2	0			3	1	0	0		
Marital status																
Single	2	50	2	3	1	0	3	5	0.5	0.06	18	21	16	10	0.02	10.05
Married	5	106	1	6	0	1	8	4			61	43	18	9		
Profession																
Formal	3	70	0	4	1	0	3	6	0.01	42.94	26	23	21	16	0,01	26.06
Informal	4	82	3	4	0	0	5	3			47	40	12	2		
Unemployed	0	4	0	1	0	1	3	0			6	1	1	1		
Educational level																
Not school	0	4	0	0	0	1	0	0	0.01	51.44	4	1	0	0	0.01	35.18
Primary	0	14	0	1	0	0	3	0			15	2	1	0		
Secondary	5	78	3	5	0	0	5	4			43	37	15	5		
University	2	60	0	3	1	0	3	5			17	24	18	14		
Previous screening																
Yes	2	51	2	4	1	0	7	4	0.7	9.62	20	15	10	70	0.19	8.76
No	5	100	1	5	0	1	4	5			56	19	9	121		

## Discussion

The prevalence of cervical precancerous lesions, which was found to be intraepithelial lesions (17%), is alarming, considering the fact that this study has been carried on “healthy” women in two of the biggest hospitals in Douala. This prevalence could be a faithful representation of this city. The predominance of high-grade lesions (5.61%) can be explained by the lack of early diagnosis and treatment; indeed, most of the participants were undergoing this examination for the very first time, with low-grade lesions found to have already evolved into high-grade lesions. Comparable findings were also reported in previous studies in Cameroun, with incidence of precancerous lesions around 7.9% [11,12]. Surprisingly, HPV 16 and 18 were not predominant among the high-risk HPV diagnosed, which could suggest a significant presence of other HR-HPVs in the Douala population. Moreover, only 3% of the HPV-positive women developed precancerous lesions, against 69% found in Yaoundé [13]. However, the most worrying fact is that these precancerous lesions (especially HSIL) were predominantly found in HPV-negative women. This could be explained by the presence of systematic dysbiosis in women with low-grade cervical neoplasia, which increases the risk of progression to high-grade cervical neoplasia.

However, 89% of the 189 women surveyed said that they had heard of cervical cancer, against the 23-39% in earlier studies performed in densely populated areas of other regions of the country, including in the Centre Region [14], South West Region [14], Northern Regions [12], and West Region [15]. This discrepancy could be explained by the fact that there is a lot of prejudice on this subject and many of them let themselves be guided by it, which has repercussions on the late completion of screening. Although both facilities are used, it became clear during the course of our study that women were reluctant to take part, so we had to extend our study in order to recruit enough female participants. This lack of commitment shows that it is part of the population health habits, as 61% of the women in our study said that they never had been screened for cervical cancer even if they were in the right age bracket to be screened, and this would also justify the predominance of high-grade lesions. The main reasons given were negligence and lack of information. However, the women who had undergone previous screening had done so mainly for medical follow-up, and only 11% had done so as a part of a campaign. This could be explained by the fact that a good number of the women in our study worked in the informal sector and therefore did not see the importance of "spending" money on an examination for which the disease had not yet been diagnosed or prescribed by a health worker. In addition, the patient-provider relationship that places the health worker as the one with all the knowledge to bring the cure to the patient favors compliance with their instructions and, above all, the performance of cervical smears. Similar findings have been found in other countries [16] who estimated that 95% of women in developing countries had never been screened, and another Ghanaian study estimated that 2.1% of Ghanaian women had undergone Pap smears. Furthermore, despite the correlation observed between the level of education and knowledge of this pathology, as a result, most of them feel unconcerned. All this contributes to the progression from low-grade to high-grade lesions. This finding highlights the need for raising the awareness of women, public health authorities, and other stakeholders about HPV role in cervical cancer development for the urgent setup of HPV vaccination campaigns in Cameroon.

Limitations

It is worth mentioning that the current study has some limitations, including its limited sample size, which may not be adequate to represent the entirety of the region. Moreover, not all types of HPV encountered by the participants during the study were characterized and were classified under the heading "others."

## Conclusions

Overall, the results of this study, which aimed to determine the prevalence of precancerous lesions and associated factors, show that the population's lack of knowledge about cervical cancer is a major cause of delayed diagnosis, hence signifying the need to intensify awareness-raising efforts. Although most of the women were in the age range required for cervical cancer screening, they had never been tested in the past. This was mainly due to a lack of information about cervical cancer, leading to the development of HSIL. The main factor associated with these lesions in the women who were included in the study was HPV infection.
